# Insights into basal cell carcinoma with bone metastasis: a comprehensive review

**DOI:** 10.1093/skinhd/vzae021

**Published:** 2025-05-09

**Authors:** Azadeh Khayyat, Mohammad Ali Esmaeil Pour, Milad Moqadam, Seyed Amir Zohouri, Amir-Reza Khalili-Toosi, Amir Behzad Heidari, Parvaneh Hatami, Bruce R Smoller

**Affiliations:** Department of Pathology, Medical College of Wisconsin, Milwaukee, WI, USA; Department of Rheumatology, Augusta University Medical College of Georgia, Augusta, GA, USA; Department of Family Medicine, BronxCare Health System, Bronx, NY, USA; Department of Biology, Simon Fraser University Faculty of Science, Burnaby, BC, Canada; Department of Pathology, Immunology and Laboratory Medicine, Rutgers New Jersey Medical School, Newark, NJ, USA; Family Medicine, Essen Health Care, Bronx, NY, USA; Department of Dermatology, Tehran University of Medical Sciences, Tehran, Iran; Department of Pathology, University of Rochester Medical Center, Rochester, NY, USA

## Abstract

Basal cell carcinoma (BCC), the most common skin cancer worldwide, is closely associated with sunlight exposure and generally exhibits a low metastatic potential, with a frequency ranging from 0.0028% to 0.55%. Despite its rarity, BCC with bony metastases causes important clinical complications. We collected information on published patients with a diagnosis of BCC with bony metastases, and examined patient demographics, tumour characteristics, histological features and treatment modalities to define patterns and outcomes. Our study encompassed 108 patients: 68 men and 40 women with a mean (SD) age of 66.9 (6.4) years. Histologically identified subtypes included 42 nodular, 28 infiltrative, 9 morphoeaform, 5 metatypical and 1 superficial BCC, with 23 patients having a mixed histopathology pattern. The main treatments were ­surgery (*n* = 98), chemotherapy (*n* = 31), immunotherapy (*n* = 16) and radiotherapy (*n* = 34). BCC with bone metastases, although rare, requires more attention due to the complexity of management. Histological subtypes such as infiltrative, sclerosing, morphoeaform, basosquamous and micronodular are associated with aggressive behaviour and the detection of symptoms such as bone pain or hypercalcaemia in patients at high risk of metastasis is important for timely diagnosis. Because of the aggressive potential and clinical implications of some subtypes, a personalized management approach with comprehensive histological and molecular profiling is essential to optimize outcomes in patients with BCC with bone metastasis.

## Teaser

Basal cell carcinoma (BCC), the most common skin cancer, typically shows low metastatic potential but causes significant clinical complications when metastatic to bone. This review examines 108 cases of BCC with metastatic bone cancer, examining patient populations, tumour characteristics, histologic subtypes and treatment. Despite the rarity of BCC with bone metastases, some histological subtypes require an individualized approach due to their aggressive nature and challenging treatment.

## Introduction

Basal cell carcinoma (BCC) is the most prevalent form of skin cancer, primarily arising from the epidermis and characterized by its potential for local invasiveness. Although BCC is commonly associated with a low rate of metastasis, rare cases of metastatic BCC have been documented, leading to significant clinical implications. Metastatic BCC is typically associated with aggressive histological subtypes, such as basosquamous carcinoma and infiltrative BCC, which may lead to metastasis to distant sites, including bone, lung and lymph nodes.^1,2^

Recent studies have reported cases of BCC with unusual metastatic patterns, including bone and visceral metastasis, often challenging the conventional understanding of its behaviour.^3,4^ A comprehensive analysis of clinical data revealed that distinct histological subtypes of BCC may exhibit varying metastatic potential, emphasizing the need for vigilant monitoring and early intervention.^5,6^

Furthermore, recent advancements in molecular biology have shed light on the genetic and molecular pathways involved in BCC progression, opening avenues for targeted therapies.^7,8^ In rare instances, BCC can metastasize to atypical sites such as the bone marrow, underscoring the necessity for heightened clinical awareness and further research into its metastatic mechanisms.^9–11^ This paper aims to explore the clinical and pathological features of metastatic BCC, contributing to a deeper understanding of its complexities and management strategies.

## Methodology

This study aimed to review and synthesize available case reports and case series regarding BCC with bone metastasis. An extensive search was performed in PubMed, Google Scholar, Scopus and Web of Science, targeting studies published to date. Keywords included ‘BCC and bone metastasis’, ‘new treatments’, ‘BCC and immunity’ and ‘BCC publishing’. The search strategy included multiple terms and medical subject headings to maximize the retrieval of relevant articles – search by keyword: [‘BCC’ AND (‘bone and bone’ OR ‘metastasis’ OR ‘new therapy’ OR ‘immunosuppression’)].

**Figure 1:**
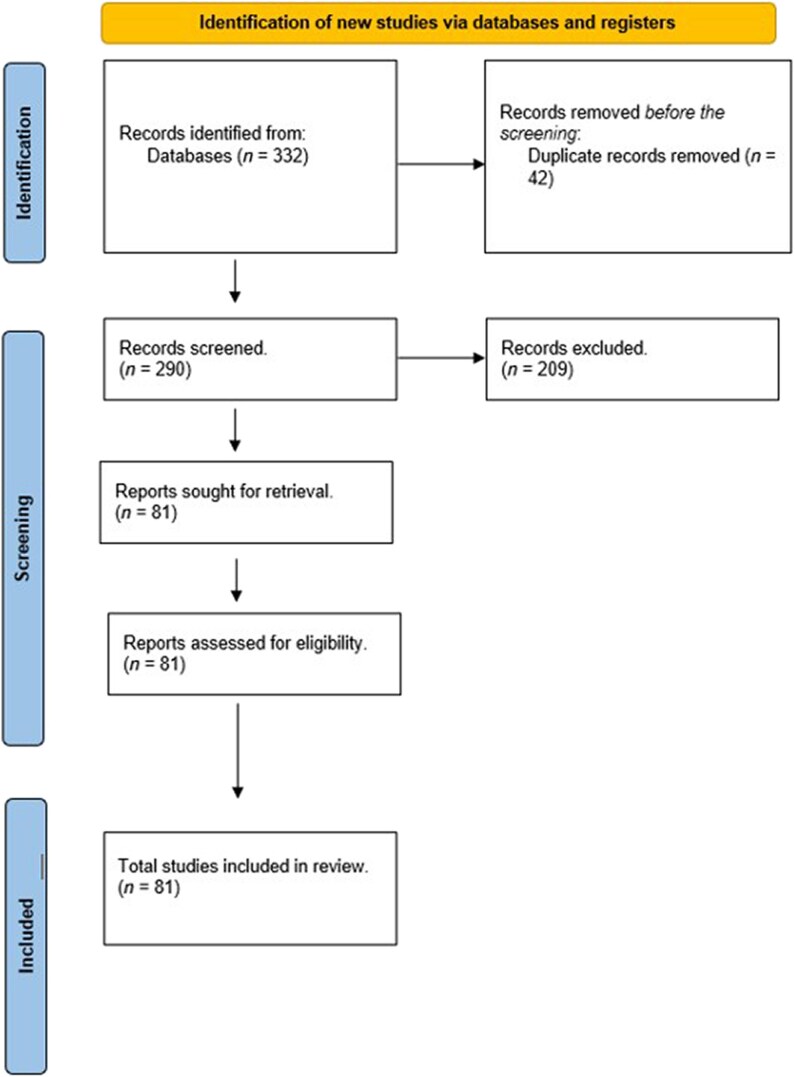
PRISMA flowchart of the study.

Following PRISMA guidelines, the initial search identified 332 articles. Title abstracts of 290 unique reports were screened after removing duplicates, of which 209 were considered not relevant to this review, and the remaining 81 full articles^1,3–5,8–84^ met the inclusion criteria. Fewer than 15 case reports highlighting these studies were included in the final analysis. A total of 108 patients with confirmed BCC bone metastases were included. This sample allowed us to build a comprehensive picture of this rare condition (Figure 1).

Data including patient demographics (age, sex), their risk factors (e.g. history of immunosuppression), tumour histological subtype (e.g. nodular and infiltrative) and pattern of metastasis (e.g. affected bone and the number of spreading metastatic sites), treatments (e.g. surgery, radiation therapy and systemic therapy) and patient outcomes (e.g. time to metastasis, survival and recurrence rate) were extracted. Descriptive statistics were used to summarize the findings.

## Review

**Figure 2:**
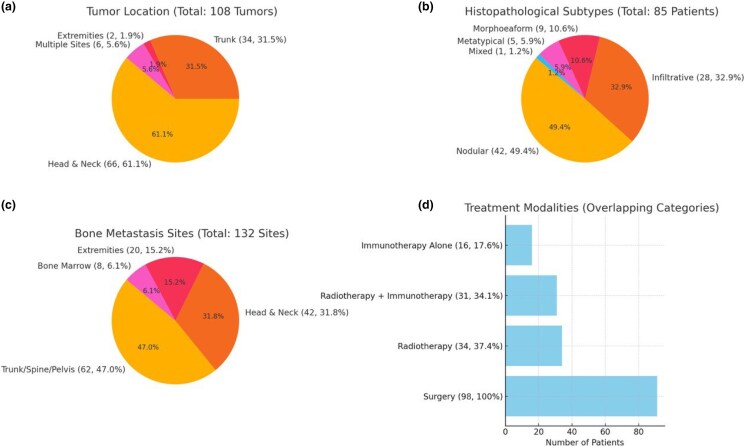
(a) Tumour location, (b) histopathological subtypes, (c) distribution of initial bone metastasis sites and (d) treatment modalities used.

Eighty-one reports of patients of BCC with bone metastasis were extracted, including 68 men and 40 women with a mean (SD) age of 66.9 (6.4) years. Most of the tumours were in the head and neck (*n* = 66), followed by the trunk (*n* = 34), the extremities (*n* = 2) and multiple sites (*n* = 6). Histologically, the lesions were classified as the nodular subtype in 42 patients, the infiltrative form in 28 and the morphoeaform in 9 patients. Five patients had a metatypical pattern. There was a mixed histopathological pattern in 23 patients, including morphoeaform and nodular subtype (*n* = 9); nodular and infiltrative (*n* = 5), metatypical and morphoeaform (*n* = 2); morphoeaform and infiltrative (*n* = 5); nodular and matricial (*n* = 1); and superficial and nodular (*n* = 1). Bone metastasis was initially in the trunk, spine or pelvis (*n* = 62), in the head and neck (*n* = 42), on the extremities (*n* = 20) and in the bone marrow (*n* = 8). Most lesions were widely spread across different body regions (Figure 2).

Many of the tumours were treated by various means. The majority of patients underwent surgery (*n* = 98), 34 patients were treated with radiotherapy, 31 with chemotherapy and 16 with immunotherapy. Most patients experienced multiple treatments. Outcomes varied: 31 patients died, 8 experienced tumour recurrence or progression, 18 survived with prevalent tumours, 18 experienced no recurrence, 5 patients deferred further treatment and 28 patients were lost to follow-up. The review highlighted the variation in outcomes and the role of appropriate treatment (Figure 2).

As every patient included in this review experienced bone metastasis, the cumulative frequency of metastasis was 100%.

The correlation between the clinical and histological characteristics of the nodular subtype of BCC and the duration of disease with bone metastases indicates important prognostic factors. In our analysis, the nodular subtype was the most prevalent (*n* = 42). Many patients presented with larger tumour sizes, specifically over 2 cm (*n* = 54), at the time of metastasis assessment. Most bone metastases originated from tumours located in the trunk and head and neck regions, with 62 cases found in the trunk, pelvis and spine. This prevalence suggests that these areas are critical for monitoring disease progression in patients with the nodular subtype. Treatment approaches were aggressive, with 98 patients undergoing surgery, reflecting a commitment to managing this subtype effectively. However, despite the generally favourable prognosis associated with the nodular subtype, the outcomes revealed that 31 patients died of disease and 8 experienced recurrence or progression, highlighting that advanced cases still pose significant management challenges. This underscores the necessity for vigilant monitoring and tailored treatment strategies for patients with nodular BCC, particularly those with bone metastases.

## Discussion

BCC, the most prevalent malignancy of the skin, represents 65–75% of all cutaneous cancers. Despite its specificity, metastasis of this tumour is very low, ranging from 0.0028% to 0.5%. Metastatic BCC was first documented in 1894 in a 46-year-old man with an acute mouth lesion, which spread to the lymph nodes of his submaxillary gland.^83^

The nodular variant is one that especially arises with chronic sun exposure and is one of the most common forms to present in sun-exposed areas such as the face and neck.

The predominance of nodular BCC in cases of bone metastasis may seem counterintuitive, given its typically less aggressive nature, but several factors could explain this pattern. Nodular BCC, although often slow growing, can reach considerable size and depth if left untreated, which increases its potential for metastasis, especially to distant sites like bone. Delayed diagnosis, particularly for nodules in less visible areas, may also give these tumours time to penetrate deeper tissues. Additionally, in immunocompromised patients, who lack the immune defences to contain tumour spread, even nodular BCC can behave more aggressively. Environmental factors, particularly prolonged ultraviolet (UV) exposure, could further accelerate genetic mutations within the tumour, pushing it toward a metastatic phenotype. Together, these factors may help explain why a subtype generally considered indolent is over-represented in cases of BCC with bone metastasis.

The nodular variants often contain mutations in *PTCH1*, induced by UV DNA damage, which was evident in the study by Huang *et al*.^84^ Such mutations result in dysregulation of the Hedgehog signalling pathway, causing uncontrolled proliferation of cells. The prevalence of *PTCH1* mutations in cases of sun-exposed BCC suggests that prolonged UV exposure is a major driving force, especially in such subtypes as nodular BCC, which develops in people with high cumulative sun exposure.

Hoashi *et al*. further highlight that this variant is typically less aggressive compared with types such as the infiltrative or morphoeaform variants, which have lower direct correlations with UV damage but instead are prone to local invasiveness. In cases where nodular BCCs progress to their advanced stages, they can develop aggressive characteristics that increase the risk of rare occurrences such as bone invasion or metastasis. This underlines the importance of early detection and intervention in sun-exposure subtypes where *PTCH1* mutations and the targeting of the Hedgehog pathway may reduce further UV exposure risk and prevent disease progression.^7^

The criteria for the evaluation of BCC with bone metastasis were originally outlined by Lattes and Kessler.^85^ This tumour has three features: firstly, the rash must originate from the skin rather than the mucosa; secondly, a metastasis location not necessarily distant from the initial lesion; and, thirdly, the similarity of primary and metastatic tumours in the histological subtype.^86^

Our review provides important information about how to diagnose and treat BCC with bone metastasis, a rare but important clinical challenge. In this review, we found that 68 men and 40 women developed BCC with bone metastasis. This is consistent with previous data, including the report by von Domarus and Stevens in which metastatic BCC was observed predominantly in men.^87^ The mean (SD) age of our cohort at the time of diagnosis of bone metastasis was 66.9 (6.4) years, which is inconsistent with the findings of a systematic analysis covering the period 1981–2011, which found a median age of 63 years (range 32–92) years at the first sign of metastasis.^88^ This finding may be due to late presentation or detection of bone metastasis which was the only metastasis included in our review (Table 1).^87,88^

**Table 1:** Summary of patient characteristics, tumour types, treatment modalities and outcomes in the 108 patients included in this review

Patient characteristics	*n*
Age (years), mean (SD)	66.9 (6.4)
Sex	
Male	68
Female	40
Predisposing factor	
Positive family history of cancer	2
History of Gorlin syndrome	6
Radiotherapy	4
**Tumour characteristics**	
Tumour size at time of metastasis	
> 2 cm	54
Primary cutaneous tumour location	
Trunk	34
Head and neck	66
Extremities	2
Multiple locations	6
Basal cell carcinoma subtype	
Metatypical/morphoeaform	5/9
Infiltrative/nodular	28/42
Superficial	1
Mixed morphoeaform and nodular	9
Mixed nodular and infiltrative	6
Mixed metatypical and morphoeaform	2
Mixed infiltrative and morphoeaform	5
Mixed nodular and matrical	1
Mixed superficial and nodular	1
Initial bone metastatic sites	
Trunk/pelvis/spine	62
Head and neck	42
Extremity	20
Bone marrow	8
Treatment	
Surgery	98
Chemotherapy	31
Immunotherapy	16
Radiotherapy	34
Unknown	6
Outcome	
Died of disease	31
Alive with disease, recurrence (recurrence or progression)	8
Alive with disease	18
Unknown/lost to follow-up	28/5
No evidence of disease	18

Data are presented as number of cases (*n*) unless otherwise stated.

The locations of the primary tumours in our analysis, with a higher number found on the head and neck (*n* = 42), are in alignment with historical studies, where these sites are frequently reported due to higher UV exposure. This association has been consistently found over decades, as supported by other analyses.^87,88^

To better support the clinical manifestations and variability in BCC metastatic presentation, we assessed, in detail, the BCC subtypes found in our review. Our results demonstrated variability in BCCs with bone metastasis histopathological subtypes, with the nodular subtype predominating, followed by infiltrative and morphoeaform subtypes, with one case being superficial BCC and several mixed patterns. Knowledge of this diversity is vital for understanding the behaviour of BCC with bone metastasis.

The nodular subtype, although generally less invasive, was more prevalent in the patients included in our review, consistent with the consensus in the literature that nodular BCC is the most common but generally less advanced type.^82^ However, the high proportion of nodular subtypes in metastatic lesions in our cohort may indicate that even less severe forms of BCCs can cause severe consequences in some cancers. Infiltrative BCC, known for its aggressive phenotype, was associated with a higher number of bone metastases in our series. This model is based on data provided by Mochel *et al.*,^89^ who noted that infiltrative BCC often shows deep tissue infiltration and is likely to recur and spread due to its growth pattern, which can avoid resection completely upon first presentation.^89^

Known for its more advanced symptoms and poorer definition in clinical presentations, morphoeaform BCC similarly demonstrated a trend toward more tissue infiltration, including extensive fibrotic reaction and perineural invasion, in our dataset, which is in agreement with the findings of Litzow *et al.*^90^

Furthermore, in our review, cases of mixed subtypes revealed difficulties in the presentation of the BCC. Awad *et al*. discussed similar complications, where mixed histological features can lead to a variety of clinical behaviours and complications in treatment, and emphasized the value of comprehensive histopathological examination to guide treatment.^16^

Although generally known to be less invasive, the presence of superficial subtypes in the metastatic setting in our cohort indicated the importance of underestimating or possibly implying the potential for metastasis, a mixture of pathological factors that was not appreciated initially. The heterogeneous pathophysiology of BCC, especially in terms of bone metastasis, highlights the challenge of clinical and histological assessment. Each subtype presents unique considerations in terms of treatment and prognosis, and includes surveillance measures regarding surgical margins, adjuvant therapy uses and monitoring of disease.

In our study, the primary sites of bone metastases – particularly the hip, pelvis and pelvis – show a strong tendency of BCC to metastasize. Previous literature found bone metastasis in 20% of cases, highlighting the severity of bone metastasis in BCC.^88^ Treatments varied in our series, with surgery being the most common approach, followed by radiotherapy, chemotherapy and immunotherapy. These findings reflected a complex approach to the management of metastatic BCC. This is in contrast to previous studies, which have reported a higher number of patients managed with chemotherapy than with radiotherapy. This difference may be responsible for the different symptoms experienced in our patients, especially bone pain.^88^ While immunotherapy is now the main treatment for most patients, chemotherapy was historically the only option available in many countries.^91^

Finally, the outcomes of our cases – 31 deaths and 8 recurrences – highlighted the poor prognosis of BCC with bone metastasis, a phenomenon that is similar to historical survival rates where median survival times often did not extend to 1-year post-metastasis diagnosis.^87^

## Conclusion

Our systematic review of BCC with bone metastasis provides insight into this unusual condition, which is more characteristic of head and neck tumours in older men. Our study demonstrated a variety of BCC subtypes, with nodular BCC predominating but more infiltrative and morphoeaform subtypes likely to spread to bone, underscoring the complexity and severity of these cases. Despite the focus on surgery combined with chemotherapy, radiotherapy and immunotherapy, prognosis remains poor, with high mortality and recurrence rates. There is a need for ongoing investigations and standardized interventions to improve outcomes in patients with metastatic BCC.

## Data Availability

The data underlying this article will be shared on reasonable request to the corresponding author.
